# Vascular plant occurrences in grasslands of Central Forest Nature Reserve (Russia): a dataset

**DOI:** 10.3897/BDJ.9.e76806

**Published:** 2021-12-10

**Authors:** Oxana V. Cherednichenko, Tatiana Gavrilova

**Affiliations:** 1 Lomonosov Moscow State University, Moscow, Russia Lomonosov Moscow State University Moscow Russia

**Keywords:** Russia, Tver Oblast, grasslands, flora, occurrence, dataset, Darwin Core

## Abstract

**Background:**

Here we present the sampling event dataset that contributes to studying the flora of grasslands in Central Forest State Nature Biosphere Reserve (part of the UNESCO World Network of Biosphere Reserves), Tver Oblast, Russia. The Reserve is located in the SW part of the Valdai Upland within the main Caspian-Baltic watershed of the Russian plain (Latitude: 56° 26' – 56° 39' N, Longitude: 32° 29' – 33° 01' E). The territory of Central Forest Reserve belongs to the subzone of subtaiga.

**New information:**

The dataset includes the occurrences of vascular plant species in four types of grasslands from 209 vegetation plots (8,506 associated occurrences), collected in 2013-2014. The dataset described in this paper has never been published before.

As the grasslands in Central Forest State Nature Biosphere Reserve are relatively unstudied, we are providing a new comprehensive dataset on the vascular plant species occurrences in the grasslands of the Reserve. The dataset contains representative information on floristic composition of plant communities in localities with assigned GPS coordinates. As the vegetation of the Reserve is typical of the subtaiga subzone, the results of analysing this dataset can be useful for grassland management in the whole subtaiga subzone.

During this study, we found one vascular plant species included in the Red Data Book of the Russian Federation, three species from the Red Data Book of Tver Oblast, as well as 10 alien vascular plant species for the Reserve. These data, especially, the occurrences of protected and alien species, contribute to our knowledge of species composition of the grasslands of the Reserve.

## Introduction

**Figure 1.**
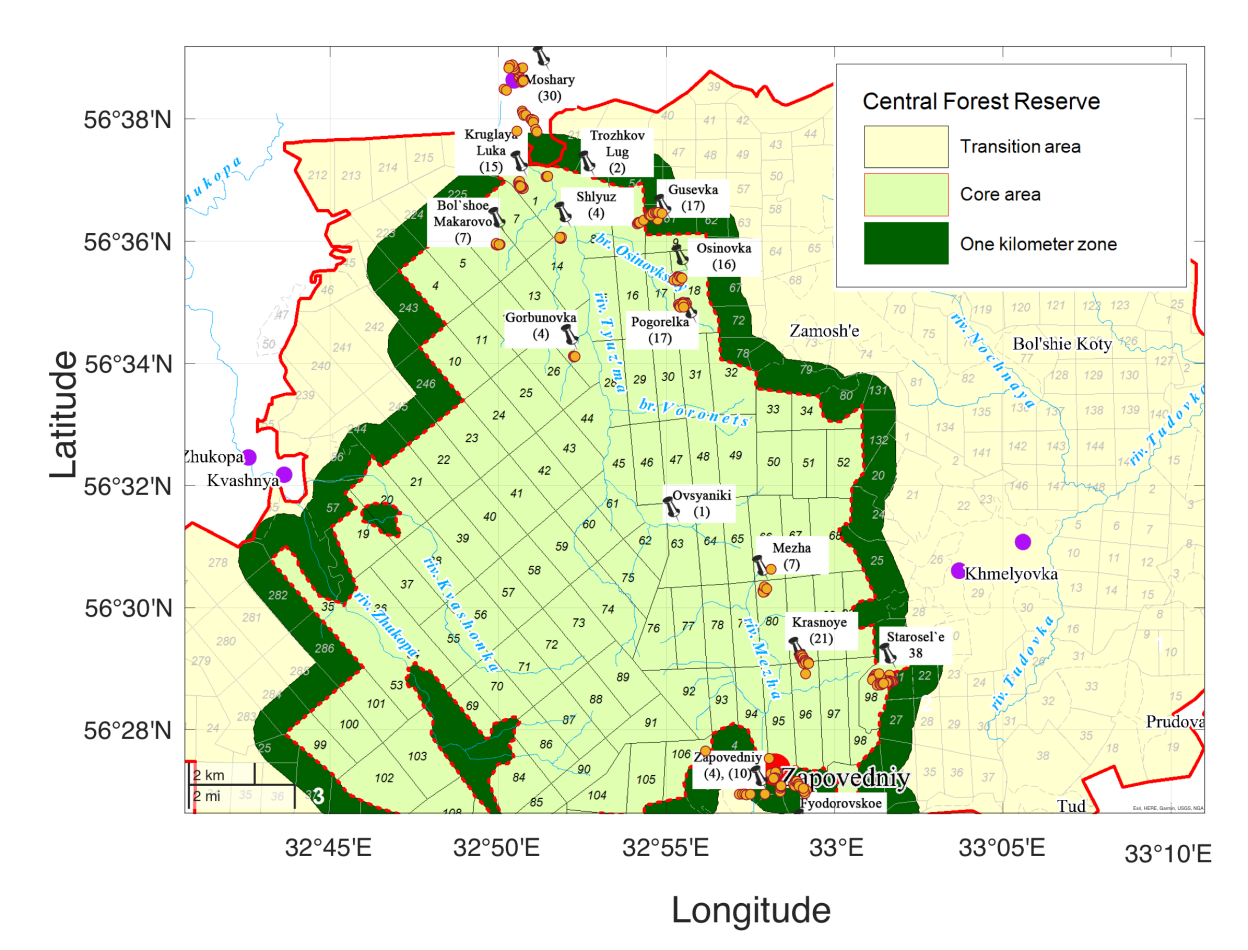
Locations of vegetation plots sampled in 2013 and 2014 in the Reserve and its vicinity.

We provide a dataset on the occurrences of vascular plants in the grasslands of Tsentral’nolesnoy Biosphere Reserve, further referred to as “Central Forest State Nature Biosphere Reserve”, as it is mentioned in GBIF. The Central Forest State Nature Biosphere Reserve (CFR) is located in the SW part of the Valdai Upland within the main Caspian-Baltic watershed of the Russian plain (Latitude: 56° 26' – 56° 39' N, Longitude: 32° 29' – 33° 01' E). CFR was established in 1931, then it was closed in 1951 and re-established in 1960. The Reserve has been a part of the UNESCO World Network of Biosphere Reserves since 1985. According to the classification of the International Union for Conservation of Nature (IUCN), the Reserve belongs to the Ia category: Strict Nature Reserve (State Nature Reserve). Like all biosphere reserves, CFR has zones free of human interference (core area), buffer zones commonly used for activities compatible with sound ecological practices, such as education and research and a transition area where restricted agricultural use is allowed. The studied grasslands are situated in the core and the transition area of the Reserve. The core area comprises 24,415 ha, the transition area 46,061 ha (Cadastral information on the Central Forest Reserve) (Fig. 1). The Reserve protects endangered species and ecosystems as a whole to maintain the biological diversity in its natural state; preserves and studies mixed broad-leaved coniferous old growth forests and raised bogs; carries out long-term environmental studies and environmental education.

The relief of the territory is mostly flat, with only low and generally gentle slopes of riverbanks and streams. The soils are sod-podzolic and gley-podzolic. The climate is humid continental (Minayeva and Shaposhnikov 1999). The mean annual rainfall for the period 1963–2014 is 760 mm (510 to 1050 mm in different years). The mean January temperature is −8.6°C (the absolute minimum is −39.4°C) and the mean July temperature is +16.9°C (the absolute maximum is +36.5°C) (Cherednichenko and Borodulina 2018).

Central Forest Reserve is situated in the subtaiga zone (Safronova et al. 2010). In the vegetation of the Reserve, forests prevail: spruce forests cover 47% of the whole area, secondary forests occupy 40%. Boggy pine forests and both oligo- and mesotrophic mires occupy 9% and 4% of the area, respectively. Grasslands cover less than 1% of the Reserve’s area (Kurayeva et al. 1999). The vegetation is typical of the taiga biome and, therefore, preserved in the Reserve as a reference.

The present-day flora of CFR includes 592 species of vascular plants (Konechnaya 2012) including 43 Red-listed species from the Red Data Book of the Russian Federation (Bardunov and Novikov 2008) and the Red Data Book of Tver Oblast (Orlov and Sokolov 2016) (40 species).

The grasslands occupy just 1% of the Reserve’s area, yet 40% of species of its vascular plant flora can be found there. In other words, although the grasslands occupy a relatively small area, they are floristically rich (Cherednichenko and Borodulina 2018).

The flora and phenology of the Central Forest Reserve were actively studied (Minayev and Konechnaya 1976, Konechnaya 2012, Shuyskaya 2018, Zorina et al. 2020), but the works describing the vegetation of the Reserve predominantly focused on the forests and the raised bogs (Karpov 1983, Kurayeva et al. 1999, Minaeva et al. 2007, Minaeva 2010, Korablev et al. 2018). Therefore, there are few publications concerning the grassland vegetation of the Reserve (Elumeeva et al. 2017, Cherednichenko and Borodulina 2018, Cherednichenko et al. 2021, [Bibr B7516172][Bibr B7516163]). Only the paper of Cherednichenko and Borodulina (2018) focused on the diversity of grasslands in the Reserve. In this data paper, we describe the dataset providing the basis for identifying four grassland types in the Reserve (managed mesic, abandoned mesic, subruderal mesic and wet grasslands); this dataset is published for the first time. The other three papers focused on functional traits of leaves (Elumeeva et al. 2017) and phytomass of two grassland types (managed mesic and abandoned mesic grasslands) (Cherednichenko et al. 2021), as well as on the decomposition rate of standard material in grassland soils (Tea bag index) (Elumeeva et al. 2021).

The present data include the sample plots made in 2013-2014 and, based on which, four types of grasslands were identified (Cherednichenko and Borodulina 2018) (Table 1). Three of these types are mesic grasslands (manaded, abandoned and subruderal) and one type belongs to wet grasslands. The sites are ecologically and physiognomically different due to their management and moisture regime. Therefore, the variability between the study sites is considerable. The sampled vegetation data will provide insight into the biodiversity and current state of the grasslands in CFR and its transition area. The dataset includes information on the occurrence of the threatened and alien species of CFR.

## General description

### Purpose

The present article is aimed at digitally representing and making available the data on the occurrences of vascular plants in the grasslands of CFR. This study is important because it was carried out in the territory where all kinds of economic activities are prohibited. As a result, the natural ecological succession has not been interrupted since 1960 and, in a number of cases, since the 1980s. So, the grasslands are being overgrown with forest, their area is decreasing and they may soon disappear completely from the territory of the Reserve and no information about them will remain. Thus, the data collected in 2013-2014 can be used in future studies to assess the characteristics of ecological succession and the restoration of natural mixed coniferous - broad-leaved forests in the Reserve area. Furthermore, these data can be used for monitoring, ecological restoration and appropriate management of the grasslands in the Reserve.

## Sampling methods

### Study extent

Grasslands (meadows, pastures and ruderal communities) in CFR exist under the protection regime in the Reserve’s core area (Fig. 1) and under agricultural use in adjacent territories. The grasslands of the core area have not been used due to the protection regime since the early 1960s and several sites since the 1980s; thus, most of the grasslands were abandoned 30–60 years ago. In the core area of CFR, there are only small patches of grasslands in the places of former settlements (villages, farmsteads, forest huts). Having been abandoned for a long time, some communities still resemble grass-forb grasslands, while others have completely changed and turned into shrub or forest vegetation. Large areas are covered with subruderal tall-herb stands dominated by forbs (*Anthriscussylvestris* (L.) Hoffm., *Chamaenerionangustifolium* (L.) Scop., *Urticadioica* L. etc.). These communities can be found in the places of abandoned housing in former villages, as well as at the sites of wild boar digging (Cherednichenko and Borodulina 2018).

The grasslands in the core area of the Reserve are not managed anymore and, therefore, are being overgrown with forest. To date, the area of grasslands has significantly decreased: in 2017, the area of the grasslands per se was estimated as 0.02% of the total Reserve area, while forest glades and wastelands covered with herbaceous vegetation occupied 0.5% of the total Reserve area (Cadastral information on the Central Forest Reserve). For example, the area of the Krasnoe site covered with grassland vegetation has decreased almost 5 times over the past 35 years (from 26.6 ha to 5.54 ha) (Cherednichenko et al. 2021).

Grasslands occupy large areas around villages in the transition area and in the one kilometre buffer zone. At present, the vast majority of these grasslands are abandoned, while only limited areas of meadows and pastures are managed (irregular mowing and low intensity grazing). The managed grasslands were studied in the vicinity of the CFR headquarters and around inhabited villages, situated in both the buffer zone and the transition area of the Reserve (Cherednichenko and Borodulina 2018).

### Sampling description

This dataset includes 209 sample plots of continental grasslands made in 2013 and 2014. In 2013, we sampled 88 plots at six sites in the south of the Reserve (Bol'shoe Fyodorovskoe, Mezha, Krasnoye, Ovsyaniki, Starosel’e, Zapovedniy). In 2014, we sampled 121 plots: 111 ones at nine sites in the north (Bol’shoe Makarovo, Gorbunovka, Gusevka, Kruglaya Luka, Moshary, Osinovka, Pogorelka, Shlyuz, Trozhkov Lug) and 10 sites in the south of the Reserve (Zapovedniy) (Fig. 1). The position of the centre of each vegetation plot was georeferenced using a Garmin GPS navigator in WGS84 datum. The dataset comprises most of the continental grasslands in the core area of CFR. However, we studied only a limited number of grassland sites in the transition zone of the Reserve due to its large area.

The size of each sample plot was 100 m^2^, which is considered appropriate for grassland vegetation (Mirkin and Naumova 2012). Plots of this size were used to sample grassland vegetation in a number of works (Chytrý and Otýpková 2003). According to Chytrý and Otýpková (2003), 16 m^2^ plots should be used as standard to sample most types of herbaceous vegetation. As we used larger sample plots (100 m^2^), we consider our vegetation samples for each plot complete in terms of their species composition. Within the sample plots, we collected the data on species composition.

Our data represent almost all grassland types in the Reserve (Table 1) according to their physiognomy and land use type. The sample plots were compiled in visually homogenous areas of vegetation along the visible gradients of the relief, as a rule from the edge to the centre of the grassland to cover the entire diversity of plant communities of each site.

We would like to highlight that the presence of two particular groups of species, namely the Red-listed species and the alien (including invasive) ones, in the dataset is closely connected with the type of the studied grasslands and their management. The alien and invasive species are provided according to Vinogradova et al. (2011), the Red-listed species follow the Red Data book of the Russian Federation (plants and fungi) (Bardunov and Novikov 2008) and the Red Data Book of Tver Oblast (Orlov and Sokolov 2016).

### Quality control

The plant species were predominantly identified in the field; when it was not possible to unambiguously identify the specimen, it was herborised for further identification at the laboratory. Most of the species were identified using the keys (Tsvelev 2000, Maevsky 2014) by Oxana Cherednichenko. In difficult cases of species, the identification was confirmed by Alexey Seregin, sedges were confirmed or identified by Yuriy Alekseev and Mikhail Kozhin and specimens of the genus Pilosella were identified by Alexander Sennikov. The collected specimens are deposited at Moscow University Herbarium (MW) and are publicly available in Moscow Digital Herbarium. In 2013, 33 herbarium specimens were collected, in 2014 - 230 herbarium specimens.

The plant scientific names in the dataset were checked against the database of TROPICOS using the iPlant Taxonomic Name Resolution Service (TNRS).

### Step description

**Figure 2.**
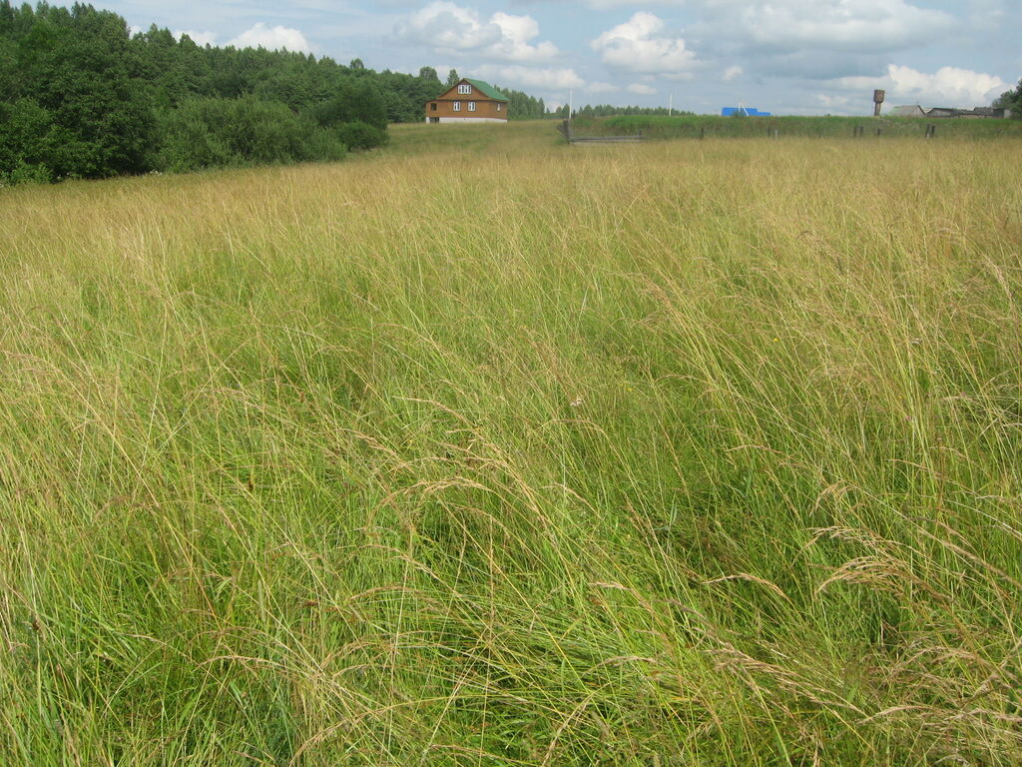
Managed mesic grassland, Bol’shoe Fyodorovskoe site, plot MW-C054 (Latitude 56.45102964 Longitude 32.97338868). Dominant species: *Carexleporina* L. and *Festucapratensis* Huds.

**Figure 3.**
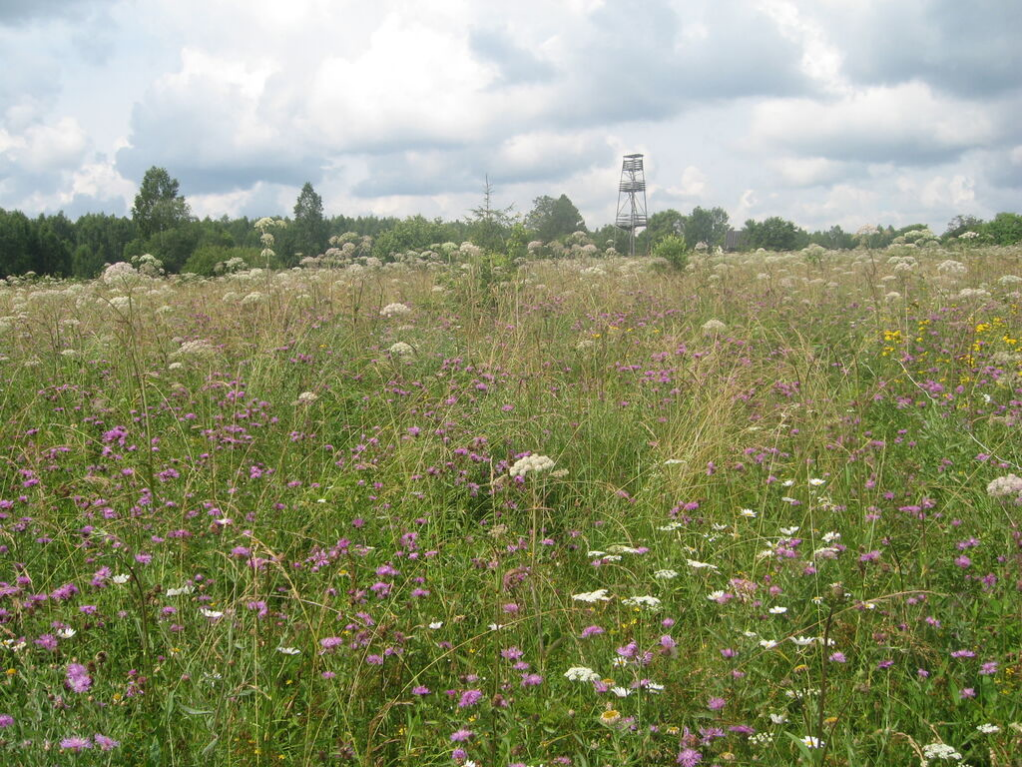
Abandoned mesic grassland, Starosel’e site, plot MW-C05 (Latitude 56.47998819 Longitude 33.02556393). Dominant species: *Angelicasylvestris* L., *Centaureaphrygia* L., *Festucapratensis* Huds., *Leucanthemumvulgare* Lam. and *Pimpinellasaxifraga* L.

**Figure 4.**
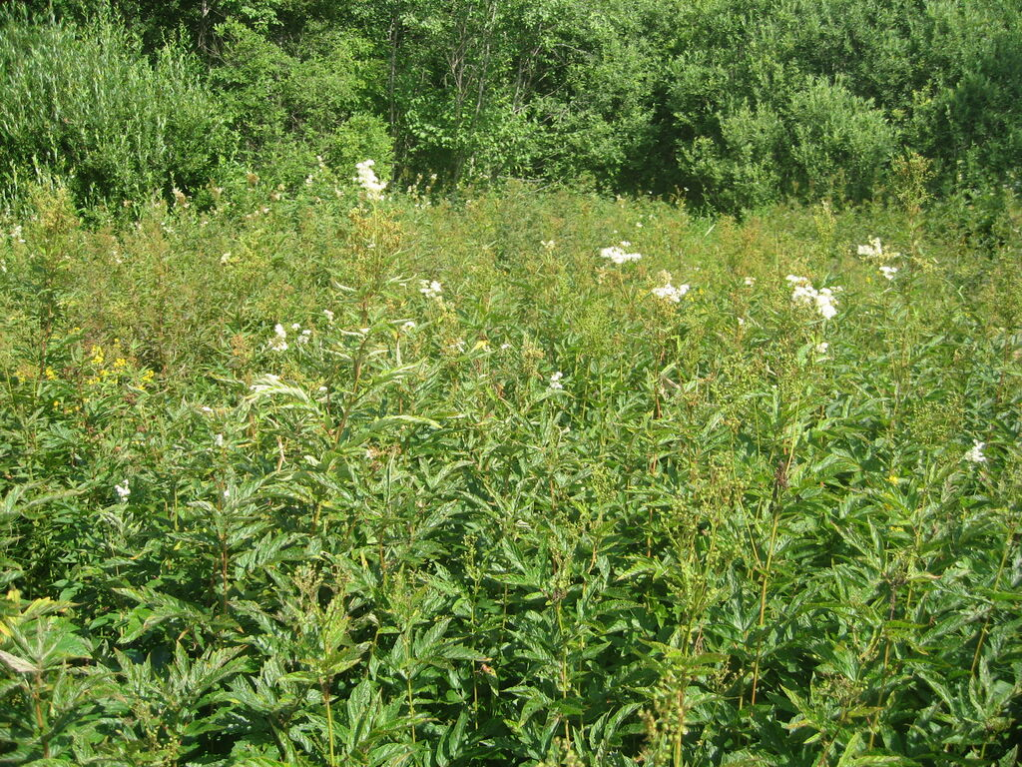
Wet grassland, site: Bol’shoe Fyodorovskoe, plot MW-C042 (Latitude 56.45129258 Longitude 32.97338742). Dominant species: *Filipendulaulmaria* (L.) Maxim. and *Lysimachiavulgaris* L.

**Figure 5.**
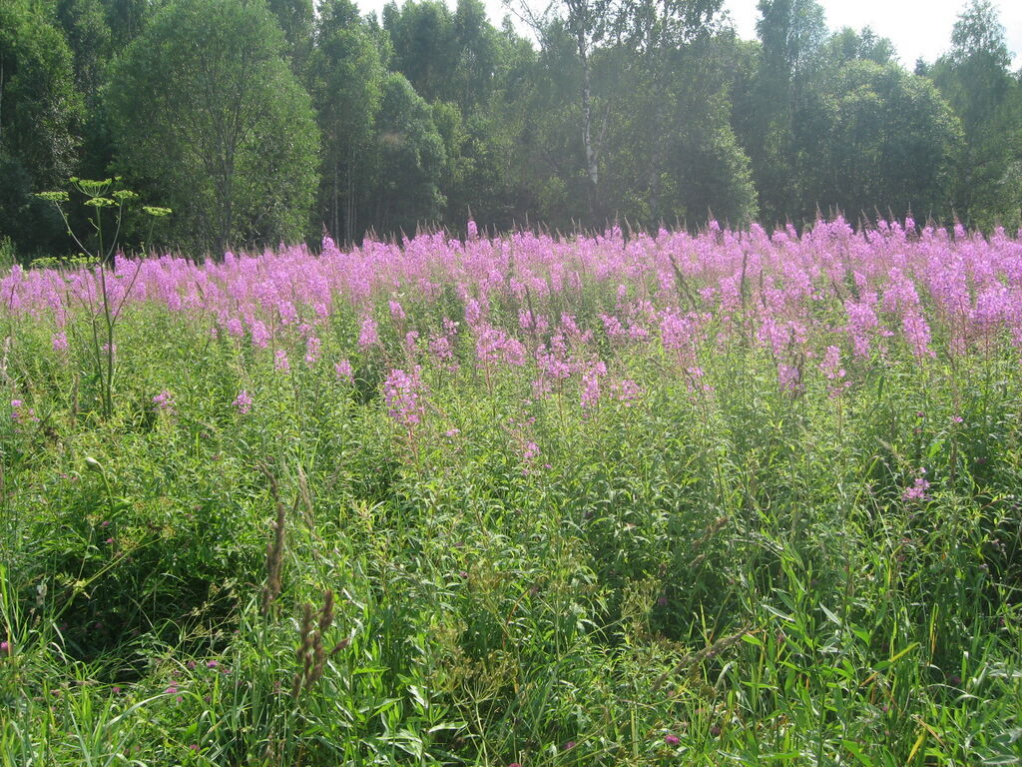
Subruderal mesic grassland, Krasnoe site, plot MW-C015 (Latitude 56.48472472 Longitude 32.98469234). Dominant species: *Anthriscussylvestris* (L.) Hoffm., *Chamaenerionangustifolium* (L.) Scop., *Dactylisglomerata* L. and *Heracleumsibiricum* L. Although the community is dominated by *Chamaenerionangustifolium* (L.) Scop., this is not a clearing, this grassland had formerly been mown and was abandoned 30 years ago. The dominance of *Chamaenerionangustifolium* (L.) Scop. may be connected with wild boar digging, as wild boars completely destroy the vegetation cover of this site once in a few years. This is a typical appearance of abandoned vegetation in the Reserve, not only at Krasnoe site, but also at a number of other sites.

As a habitat characteristic, we used the classification of grassland types described in Cherednichenko and Borodulina (2018). The grassland types were determined using cluster analysis, indicator species analysis and phytoindication assessment. Thus, we distinguish four types of grasslands: managed mesic (Fig. 2), abandoned mesic (Fig. 3), wet (Fig. 4) and subruderal mesic grasslands (Fig. 5), that are presented in Table 1; they are also available in the GBIF dataset (Cherednichenko 2021).

These four grassland types differ in management, floristic composition and ecological conditions, as well as in the proportion of coenotic and functional groups (including forbs, graminoids and woody species). Managed mesic grasslands (Fig. 2) are communities with the dominance of grasses and forbs under moderate grazing and irregular mowing. Their indicator species are *Cynosuruscristatus* L., *Leontodonautumnalis* L., *Plantagomajor* L., *Potentillaanserina* L. and *Taraxacumofficinale* Wigg. Abandoned mesic grasslands (Fig. 3) were mown or grazed in the past and they still resemble typical meadows with the dominance of forbs and grasses. Their indicator species are *Hieraciumumbellatum* L., *Potentillaerecta* (L.) Raeusch., *Rumexacetosa* L., *Trolliuseuropaeus* L. and *Violacanina* L. Wet grasslands are tall-herb meadowsweet communities (Fig. 4), forming in small relief depressions and along temporary streams, probably in the places of abandoned hayfields. Their indicator species are *Cirsiumpalustre* (L.) Scop., *Crepispaludosa* (L.) Moench, *Filipendulaulmaria* (L.) Maxim., *Galiumpalustre* L. and *Violapalustris* L. Subruderal mesic grasslands (Fig. 5) are not currently managed and are totally covered with ruderal and nitrophilous species. Their indicator and dominant species are *Anthriscussylvestris* (L.) Hoffm., *Chamaenerionangustifolium* (L.) Scop., *Cirsiumarvense* (L.) Scop., *Dactylisglomerata* L. and *Urticadioica* L.

Table 1 shows that abandoned and subruderal mesic grasslands were the most widespread in the study area, while wet grasslands, associated with specific, more humid conditions, were less frequent. Managed mesic grasslands were less widespread, since there are few inhabited villages in the transition area of the Reserve.

## Geographic coverage

### Description

Tver Oblast, Russia

### Coordinates

56.44882 and 56.64804 Latitude; 32.83221 and 33.02906 Longitude.

## Taxonomic coverage

### Description

The dataset includes 261 unique scientific names of vascular plants (260 taxa were identified to species ranks and one taxon to aggregate rank only – *Alchemillavulgaris* agg.). General taxonomic coverage is one phylum, four classes, 48 families, 154 genera and 261 species of vascular plants.

Thus, the dataset comprises 44.1% of the whole Reserve's flora, which consists of 592 species (Konechnaya 2012). Furthermore, the flora of the studied grasslands makes up 16.5% of the Tver Oblast checklist (1579 species) (Notov 2005).

There are 190 species typical of the grasslands per se in the list compiled by Konechnaya (2012) (the total number of species in this list is 529), while our dataset contains 261 species. A total of 141 species were included both in the list provided by Konechnaya (2012) and in our dataset, the rest of the species from our dataset are listed in Konechnaya (2012) as characteristic of forests and forest edges, as well as of ruderal and wet habitats. Five species that we recorded, namely *Hieraciumscandinavicum* Dahlst., *Potentillaintermedia* L., *Swidaalba* (L.) Opiz, *Triticumaestivum* L. and *Viciavillosa* Roth, are absent in the list compiled by Konechnaya (2012), all these species having low occurrence in the transition zone of the Reserve.

Revealing the complete grassland flora of the Reserve was beyond the scope of our study. The dataset we published is based on sampling the vegetation of particular grassland sites in the Reserve. Therefore, we detected less species than had previously been recorded (Konechnaya 2012) for the grasslands of the Reserve. Nevertheless, we provide the geographical coordinates for all the grassland species that we observed.

During our grassland studies, we found one vascular plant species included in the Red Data Book of the Russian Federation (Bardunov and Novikov 2008): *Dactylorhizabaltica* (Klinge) Nevski and three Red-listed species from the Red Data Book of Tver Oblast (Orlov and Sokolov 2016): *Coeloglossumvirid*e (L.) С. Hartm., *Gymnadeniaconopsea* (L.) R. Br. and *Salixphylicifolia* L.

Ten alien species were recorded in the dataset, including eight invasive plants: *Conyzacanadensis* (L.) Cronq., *Epilobiumciliatum* Rafin., *Festucaarundinacea* Schreb., *Juncustenuis* Willd., *Lepidothecasuaveolens* (Pursh) Nutt., *Loliumperenne* L., *Malusdomestica* Borkh. and *Petasiteshybridus* (L.) Gaertn., B. Mey. & Scherb. (according to the list of Vinogradova et al. (2011)); and two cultivated species: *Swidaalba* (L.) Opiz and *Triticumaestivum* L.

Vinogradova et al. (2011) list 100 invasive species for Tver Oblast and 39 species for Nelidovsky District, amongst them, species with low activity prevail. In general, the activity of the invasive flora in Nelidovsky District is low (Vinogradova et al. 2011). Invasive species were seldom observed at the study sites, occurring mostly in managed mesic grasslands and were completely absent in wet ones (Cherednichenko and Borodulina 2018). *Juncustenuis* Willd. (35%) was the most widespread in managed mesic grasslands and *Malusdomestica* Borkh. (32%) in abandoned mesic grasslands. The list of Vinogradova et al. (2011) does not include two alien species cultivated in the transition area of the Reserve which were observed at the study sites (*Triticumaestivum* L. and *Swidaalba* (L.) Opiz).

### Taxa included

**Table:** 

Rank	Scientific Name	
kingdom	Plantae	
phylum	Tracheophyta	
class	Liliopsida	
class	Magnoliopsida	
class	Pinopsida	
class	Polypodiopsida	
family	Amaranthaceae	
family	Apiaceae	
family	Asteraceae	
family	Athyriaceae	
family	Betulaceae	
family	Boraginaceae	
family	Brassicaceae	
family	Campanulaceae	
family	Caprifoliaceae	
family	Caryophyllaceae	
family	Convolvulaceae	
family	Cornaceae	
family	Crassulaceae	
family	Cupressaceae	
family	Cyperaceae	
family	Dryopteridaceae	
family	Equisetaceae	
family	Euphorbiaceae	
family	Fabaceae	
family	Geraniaceae	
family	Hypericaceae	
family	Juncaceae	
family	Lamiaceae	
family	Linaceae	
family	Malvaceae	
family	Onagraceae	
family	Onocleaceae	
family	Ophioglossaceae	
family	Orchidaceae	
family	Orobanchaceae	
family	Pinaceae	
family	Plantaginaceae	
family	Poaceae	
family	Polemoniaceae	
family	Polygalaceae	
family	Polygonaceae	
family	Primulaceae	
family	Ranunculaceae	
family	Rhamnaceae	
family	Rosaceae	
family	Rubiaceae	
family	Salicaceae	
family	Saxifragaceae	
family	Scrophulariaceae	
family	Solanaceae	
family	Urticaceae	
family	Viburnaceae	
family	Violaceae	

## Temporal coverage

### Notes

Data range: 08.07.2013 - 18.07.2013; 05.07.2014 - 21.07.2014.

## Usage licence

### Usage licence

Creative Commons Public Domain Waiver (CC-Zero)

## Data resources

### Data package title

Vascular plants of grasslands in Central Forest Nature Reserve (Tver Oblast, Russia).

### Resource link

https://doi.org/10.15468/ufbxsn

### Alternative identifiers

https://www.gbif.org/dataset/bc6d4ac6-a710-456e-b75f-8dbaf540eab3

### Number of data sets

1

### Data set 1.

#### Data set name

Vascular plants of grasslands in Central Forest Nature Reserve (Tver Oblast, Russia).

#### Number of columns

44

**Data set 1.** 

Column label	Column description
occurrenceID	An identifier for the occurrence (unique). For example, "urn:lsid:biocol.org:col:15550:14:M102:3025".
dcterms:type	The nature or genre of the resource. A constant ("Dataset").
dcterms:modified	The most recent date-time on which the resource was changed. A constant ("2021-10-12").
dcterms:language	A language of the resource. A constant ("en" = English).
dcterms:license	A legal document giving official permission to do something with the resource. A constant (http://creativecommons.org/licenses/by/4.0/legalcode).
dcterms:rightsHolder	A person or organisation owning or managing rights over the resource. A constant ("Moscow State University").
dcterms:accessRights	Information about who can access the resource or an indication of its security status. A constant ("Use under CC BY 4.0").
institutionID	An identifier for the institution having custody of the object(s) or information referred to in the record. A constant (http://grbio.org/institution/moscow-state-university for the Moscow State University).
collectionID	An identifier for the collection or dataset from which the record was derived. A constant ("urn:lsid:biocol.org:col:15550" for the Moscow University Herbarium).
datasetID	An identifier for the set of data. May be a global unique identifier or an identifier specific to a collection or institution. A constant ("urn:lsid:biocol.org:col:15550:15").
institutionCode	The name (or acronym) in use by the institution having custody of the object(s) or information referred to in the record. A constant ("Moscow State University").
datasetName	The name identifying the dataset from which the record was derived. A constant ("Vascular plants of grasslands in Central Forest Nature Reserve (Tver Oblast, Russia)").
ownerInstitutionCode	The name (or acronym) in use by the institution having ownership of the object(s) or information referred to in the record. A constant ("Moscow State University").
basisOfRecord	The specific nature of the data record. A constant ("HumanObservation").
informationWithheld	Additional information that exists, but that has not been shared in the given record. A constant ("Associated ecological data, voucher information, syntaxa, functional traits, geomorphological features of plots, id of plots").
recordedBy	A list (concatenated and separated) of names of people, groups or organisations responsible for recording the original occurrence. A variable, for example "Oxana V. Cherednichenko | Valentina P. Borodulina | Veronika V. Gorik".
occurrenceStatus	A statement about the presence or absence of a taxon at a location. A constant ("present").
eventDate	The date or interval during which an event occurred. For occurrences, this is the date when the event was recorded. A variable.
habitat	A category or description of the habitat in which the Event occurred. For example, "managed mesic meadows".
higherGeography	A list (concatenated and separated) of geographic names less specific than the information captured in the locality term. A constant ("Europe | Russian Federation | Tver Oblast").
continent	The name of the continent in which the location occurs. A constant ("Europe").
country	The name of the country or major administrative unit in which the location occurs. A constant ("Russian Federation").
countryCode	The standard code for the country in which the location occurs. A constant ("RU").
stateProvince	The name of the next smaller administrative region than country (state, province, canton, department, region etc.) in which the location occurs. A constant ("Tver Oblast").
county	The full, unabbreviated name of the next smaller administrative region than stateProvince (county, shire, department etc.) in which the Location occurs. For example, "Andreapol’skiy District".
verbatimLocality	The original textual description of the place. A variable with grid square index. For example, "Pogorelka".
decimalLatitude	The geographic latitude (in decimal degrees, using the spatial reference system given in geodeticDatum) of the geographic centre of a location. A variable.
decimalLongitude	The geographic longitude (in decimal degrees, using the spatial reference system given in geodeticDatum) of the geographic centre of a location. A variable.
geodeticDatum	The ellipsoid, geodetic datum or spatial reference system (SRS) upon which the geographic coordinates given in decimalLatitude and decimalLongitude are based. A constant ("WGS84").
coordinateUncertaintyInMeters	The horizontal distance (in metres) from the given decimalLatitude and decimalLongitude describing the smallest circle containing the whole of the location. A constant ("8").
coordinatePrecision	A decimal representation of the precision of the coordinates given in the decimalLatitude and decimalLongitude. A constant ("0,00001").
georeferencedBy	A list (concatenated and separated) of names of people, groups or organisations who determined the georeference (spatial representation) of the location. For example, "Oxana V. Cherednichenko | Valentina P. Borodulina | Veronika V. Gorik".
georeferencedDate	The date on which the Location was georeferenced. A variable.
georeferenceSources	A list (concatenated and separated) of maps, gazetteers or other resources used to georeference the Location, described specifically enough to allow anyone in the future to use the same resources. A constant ("field GPS data").
scientificName	The full scientific name, with authorship and date information, if known. A variable, for example, "*Achilleamillefolium* L.".
kingdom	The full scientific name of the kingdom in which the taxon is classified. A constant ("Plantae").
phylum	The full scientific name of the phylum or division in which the taxon is classified. A constant ("Tracheophyta").
family	The full scientific name of the family in which the taxon is classified. For example, "Asteraceae".
genus	The full scientific name of the genus in which the taxon is classified. For example, "*Achillea*".
SpecificEpithet	The name of the first or species epithet of the scientificName. For example, "*millefolium*".
taxonRank	The taxonomic rank of the most specific name in the scientificName. A constant ("Species").
scientificNameAuthorship	The authorship information for the scientificName formatted according to the conventions of the applicable nomenclaturalCode. For example, "L.".
nomenclaturalCode	The nomenclatural code (or codes in the case of an ambiregnal name) under which the scientificName is constructed. A constant ("International Code of Nomenclature for algae, fungi and plants").
taxonomicStatus	The status of the use of the scientificName as a label for a taxon. A constant ("accepted"). The taxonomy is linked to a checklist dataset (https://doi.org/10.15468/7zk2y5) that defines the concept.

## Figures and Tables

**Table 1.** Grassland types distinguished in the dataset and their distribution amongst the studied sites.

**Grassland type**	**Number of sample plots**	**Number of sites**
Managed mesic grasslands	51	3
Abandoned mesic grasslands	79	10
Wet grasslands	24	6
Subruderal mesic grasslands	55	10
